# A comparative study of green solid contact ion selective electrodes for the potentiometric determination of Letrozole in dosage form and human plasma

**DOI:** 10.1038/s41598-023-47240-3

**Published:** 2023-11-18

**Authors:** Sherin M. Alqirsh, Nancy Magdy, Maha F. Abdel-Ghany, Noha F. El Azab

**Affiliations:** Pharmaceutical Analytical Chemistry Department, Faculty of Pharmacy, Ain Shams University, Organization of African Unity Street, Abasia, Cairo, 11566 Egypt

**Keywords:** Chemistry, Nanoscience and technology

## Abstract

Analysis of drugs clinically and their identification in biological samples are of utmost importance in the process of therapeutic drug monitoring, also in pharmacokinetic investigations and tracking of illicit medications. These investigations are carried out using a variety of analytical methods, including potentiometric electrodes. Potentiometric electrodes are a wonderful solution for researchers because they outperform other methods in terms of sustainability, greenness, and cost effectiveness. In the current study, ion-selective potentiometric sensors were assembled for the aim of quantification of the anticancer drug Letrozole (LTZ). The first step was fabrication of a conventional sensor based on the formation of stable host–guest inclusion complex between the cationic drug and 4-tert-butylcalix-8-arene (TBCAX-8). Two additional sensors were prepared through membrane modification with graphene nanocomposite (GNC) and polyaniline (PANI) nanoparticles. Linear responses of 1.00 × 10^–5^–1.00 × 10^–2^, 1.00 × 10^–6^–1.00 × 10^–2^ and 1.00 × 10^–8^–1.00 × 10^–3^ with sub-Nernstian slopes of 19.90, 20.10 and 20.30 mV/decade were obtained for TBCAX-8, GNC, and PANI sensors; respectively. The developed sensors were successful in determining the drug LTZ in bulk powder and dosage form. PANI modified sensor was used to determine LTZ in human plasma with recoveries ranging from 88.00 to 96.30%. IUPAC recommendations were followed during the evaluation of the electrical performance of the developed sensors. Experimental conditions as temperature and pH were studied and optimized. Analytical Eco-scale and Analytical GREEness metric were adopted as the method greenness assessment tools.

## Introduction

Women are the core of any family and are valuable contributors to the society. Their physical and mental health is always of great concern^1^. Common problems that affect a large percentage of women around the world are polycystic ovary syndrome and breast cancer. Polycystic ovary syndrome (PCOS) is an endocrine abnormality that affects a woman’s ovaries. It is one of the leading causes of anovulatory infertility in women of reproductive age. About 1 of every 10 women of this age group has PCOS, greatly impacting their quality of life^2^. The most prevalent type of cancer among women is breast cancer, listed second among causes for cancer related mortality in women^3^.

**Figure 1:**
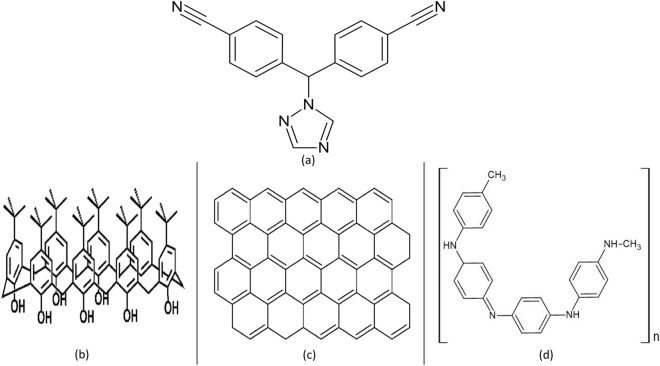
Chemical structure of (**a**) Letrozole, (**b**) TBCAX-8, (**c**) graphene, and (**d**) polyaniline.

Letrozole (LTZ), chemically known as 4,4′-((1H-1,2,4-triazol-1-yl)methylene) dibenzonitrile (Fig. 1), is a selective non-steroidal aromatase inhibitor used in management of breast cancer and PCOS^2,4^. Aromatase catalyzes the final step of estrogen biosynthesis, thus inhibiting this enzyme means reduced estrogen levels and reduced negative feedback on the hypothalamic pituitary axis resulting in reduced growth of hormone dependent cancer cells and increased endogenous follicle stimulating hormone (FSH) release^5^. LTZ was proved to enhance live birth and lower time to conception, and was recommended to be used as the first line ovulation inducing agent (superior to clomiphene citrate) for women with PCOS and infertility^6^.

The recommended dose of LTZ is 2.5 mg once a day. A steady state concentration of 107 ng/mL^7^ is reached within 2–6 weeks of drug administration, unaffected by food. LTZ is mainly eliminated in urine as an inactive glucuronide metabolite, only 6% unchanged LTZ are recovered in urine. Letrozole proved to be of high potency compared to other aromatase inhibitors^8^. Generally, oral anti-hormonal drugs express interpatient variability in their pharmacokinetics. So, therapeutic drug monitoring is essential for dosage adjustment and prevention of under or overdosing^9^.

Quantification of LTZ in different dosage forms and biological samples has been previously reported utilizing various analytical techniques including chromatography^10–18^, spectrophotometry^19–21^, potentiometry^22,23^, and voltammetry^24–26^.

Ion-selective electrodes (ISEs) have been widely used as chemical sensors for over a century with an increasing range of applications and sensed chemical species. Potentiometric detection using ISEs offers the advantages of being simple, fast, reasonably selective, non-destructive, and environmentally benign. Also, readings of good sensitivity and consistency are produced at affordable prices, which make them highly adoptable as rapid ion detectors in many fields including biomedical, industrial, agricultural, and environmental analyses^27–30^. One more attractive feature is that measurements are unaffected by color or turbidity, this opened the door for the application of ISEs in food analysis^31,32^. Recently, the application of ISEs in pharmaceutical tracing in various matrices has been embraced as a better alternative method. Additionally, ISEs are portable for in-situ monitoring due to their straightforward design and utilization of small volumes of material^33–35^.

ISEs were initially designed as liquid contact electrodes, but their responses were unreliable, and their lifespan was short. As a result, solid contact electrodes were then developed. However, these solid phase electrodes, including coated wire and glassy carbon electrodes, had several drawbacks, such as the development of water films internally. Accordingly, the surface of the electrode is altered and the electron transmission rate across it slows^36^. Membrane electrodes have experienced significant enhancement and modification in recent years for improved response behaviors^35,37^. The use of conductive polymers (CPs), either as a part of the ion-selective membrane or as an integrated layer between the ion-selective membrane and the electrode is one of the innovations made to address these issues. The incorporation of CPs as ion-to-electron conductivity transducers has shown impressive outcomes including reduced signal drifts, decreased detection limits, and improved potential stability over the long run^38,39^. Solid contact electrodes have been enhanced using multiwall carbon nanotubes (MWCNT) to give better signal quality and electrode stability. The use of nanotechnology in preparing modified electrochemical sensors has showed outstanding results involving improved selectivity and sensitivity^33,40^. TBCAX-8 forms stable host–guest inclusion complexes through dipole–dipole interaction with many cationic compounds, therefore it is commonly used as ionophore^41^.

Graphene nanocomposite (GNC) has some special physicochemical properties, particularly good electrical conductivity, and high surface area. The hydrophobic character of graphene helped in preventing the development of a water layer between the solid contact electrode and the ion-selective membrane, thus it is frequently used in fabrication of electrochemical sensors as a transducing material^42,43^.

Polyaniline (PANI) is a conductive polymer that has attracted much attention due to its high conductivity and unique properties^44^. The use of nano sized particles was proved to increase the electrical conductivity of PANI^45^.

Potentiometric sensors for quantification of LTZ have been previously reported in literature, PVC and carbon paste electrodes based on ion pair between letrozole and sodium tetraphenyl borate were utilized for LTZ determination in pharmaceutical formulations^22^. Modified magnesium and copper oxide nanoparticles were used to prepare coated wire membrane sensors that were used for quantification of LTZ in pharmaceutical formulations and human plasma^23^.

The objective of the present study is the establishment of novel sensors utilizing graphene nanocomposite and polyaniline nanoparticles as green, simple, and cost-effective electrodes for the quantitation of LTZ in pharmaceutical formulations, bulk powder, and biological fluids after studying their sensitivity, response time and shelf life.

## Experimental

### Materials and reagents

All chemicals used in this work were of analytical grade, and the water used was distilled twice. Sodium tetraphenylborate (NaTPB), high molecular weight polyvinyl chloride (PVC) were purchased from Fluka (Steinheim, Germany). Graphene, di-octyl phthalate (DOP), TBCAX-8, tetrahydrofuran (THF) and acetonitrile were obtained from Sigma Aldrich (Steinheim, Germany). Ammonium persulfate (APS) was supplied by Oxford (Maharashtra, India). Sodium dodecyl sulfate (SDS) and aniline were purchased from Merck (Dermstadt, Germany). Hydrochloric acid, sodium hydroxide, starch, lactose, and xylene were supplied by El Nasr Pharmaceuticals Co., (Cairo, Egypt). Letrozole (LTZ) pure standard was supplied by Hikma specialized pharmaceuticals, Cairo, Egypt. Pharmaceutical formulation: Femara® tablets (2.5 mg/tablet) was produced by Novartis, Switzerland. Human plasma was obtained from ‘Vacsera’ (Giza, Egypt) with agreement.

### Instrumentation

For potentiometric measurements, a Thermo Scientific Orion Ag/AgCl double-junction reference electrode (model 900200, USA), and a Jenway digital ion analyzer (model 3330, UK) were in use. UV–VIS double-beam spectrophotometer (Shimadzu 1601 PC; Kyoto, Japan) was used for spectrophotometric characterization of polyaniline nanoparticles. Particle size estimation was performed using Nano-ZS (Malvern, UK).

Jenway pH glass electrode (model 924005-BO3-Q11C, Essex, UK) was utilized for pH adjustments.

### Standard solutions

## LTZ standard stock solution (1.00 × 10^–2^ M)

0.2853 g of letrozole was weighed and dissolved in 100 mL 1:4 HCl solution.

## LTZ standard working solutions (1.00 × 10^–8^ to 1.00 × 10^–2^ M)

Preparation of working solutions was made through proper dilution of the stock solution with water according to calculated volumes to prepare each solution.

### Procedure

#### Graphene nanocomposite (GNC) preparation

The solution dispersion method was used for preparation of 10% (w/w) graphene nanocomposite in a tube. 10.00 mg of graphene powder was weighed and dispersed in 1.00 mL xylene then sonicated for 5 min. In another tube, 95.00 mg of PVC was dissolved in 3.00 mL THF followed by the addition of 0.20 mL DOP. Finally, the contents of both tubes were mixed and sonicated for 10 min^46^.

#### Polyaniline nanoparticles (PANI) preparation and characterization

Micellar emulsion chemical polymerization technique was performed^47^ using SDS as the surfactant. 50.00 mL of water was added to a round-bottomed flask along with equimolar amounts (1.30 M) of aniline (5.95 ml) and SDS (18.75 g), the mixture was mechanically stirred for one hour where a milky white solution was formed. To this solution, 50.00 mL of APS (1.30 M) was added slowly dropwise. After 2.50 h of polymerization, a dark green dispersion was produced. The temperature was set to 20 °C throughout the whole process using a thermostated bath.

To purify the produced PANI dispersion, dialysis was performed against deionized water for 48 h using a dialysis membrane (12,000 Da, Sigma). Following that, centrifugation at 15,000 rpm was carried out for 10 min and the dispersion was washed four times using distilled water. Dispersion in xylene (10% v/v) was made and kept in an amber glass bottle.

UV–VIS spectroscopy was used to characterize the nanoparticles through their absorbance spectrum. Additionally, dynamic light scattering (DLS) was used in measuring their particle size.

#### Ion-selective membrane sensors’ fabrication

The membrane solution was prepared as follows: 0.035 g of TBCAX-8, 0.01 g NaTPB, and 0.35 mL of DOP plasticizer were mixed in a small petri dish. 0.19 g of PVC was then added, and 6.00 mL THF was used to dissolve the mixture.

The body of electrode consists of a polyethylene tube in which graphite rod (15 mm in length and 5 mm in diameter) was inserted with mechanically polished protruding end. The body was internally filled with liquid mercury and a 1 mm Ag/AgCl wire was placed inside it.

30.00 µL of the ion-selective membrane (ISM) solution was drop-casted on the surface of the graphite rod and then left overnight at room temperature until complete dryness (Sensor 1).

A GNC modified electrode was prepared where 20.00 µL of graphene nanocomposite was drop-casted on a graphite rod’s surface and left to dry. The ISM layer was then drop-casted and dried (Sensor 2).

Another modified electrode was fabricated (PANI sensor) where 10.00 µL of polyaniline nanoparticles (PANI NPs) was drop-casted on a graphite rod followed by adding the ISM layer after drying (Sensor 3).

All sensors were conditioned in a 1.00 × 10^–3^ M solution of LTZ overnight. When the sensors are not in use, they are stored in the same conditioning solution.

#### Calibration of fabricated sensors

For calibration, LTZ solutions covering the ranges of 1.00 × 10^–8^–1.00 × 10^–2^ M were prepared for the fabricated sensors. 50.00 mL of each solution was analyzed separately using fabricated sensors. The measured EMF values were plotted as a function of − log [LTZ]. The linear part of the curve was used to compute the regression equation for each sensor. Unknown concentrations of LTZ were calculated using these equations.

#### Optimization of working conditions

##### pH effect

The impact of changing pH values on the potential was evaluated. pH values ranging from 1 to 9 at pH intervals of 0.5 were studied. Concentration levels of 1.00 × 10^–3^ and 1.00 × 10^–4^ M were used. pH adjustments were made using 0.10 N NaOH and HCl solutions. The dependence of the electrode potential on pH value was plotted for each sensor.

##### Temperature effect

Solutions covering the concentration ranges of 1.00 × 10^–5^–1.00 × 10^–2^, 1.00 × 10^–6^–1.00 × 10^–2^, and 1.00 × 10^–8^–1.00 × 10^–3^ for sensors 1, 2, and 3; respectively were prepared. Potential was recorded at 25, 30, 35, 40 and 45 °C for each sensor separately. The impact of temperature on the potential of each sensor was plotted with the temperature values on the x-axis and the potential readings on y-axis.

##### Selectivity

Many interfering substances as inorganic cations (Na^+^, K^+^, NH_4_^+^, Ca^++^, Mg^++^), sugars (lactose, starch) and some co-administered drugs (clomiphene citrate (CLO), tamoxifen (TAM), and metformin HCl (MFH)) were evaluated using the separate solution method, where a sensor’s potential is measured in two different solutions having the same ion activity; A (LTZ ions) and B (interfering ion) where a_A_ = a_B_. The re-arranged Nicolsky-Eisenman equation was used to calculate the selectivity coefficients as follows:

$${\text{Log}}_{{}}\,K^{pot}_{A,B} = \left[ {\left( {E_{B} - E_{A} } \right)/S + \left( {{1} - Z_{A} /Z_{B} } \right)} \right] \times {\text{Log}}\,a_{A}$$

where *K*^*pot*^_*A,B*_: potentiometric selectivity coefficient. *E*_*A*_: electrode potential on measuring 1.00 × 10^–3^ M LTZ. *E*_*B*_: electrode potential on measuring 1.00 × 10^–3^ M interferent. *S*: slope of the calibration curve. *Z*_*A*_, *Z*_*B*_: charge of LTZ and interferent; respectively. *a*_*A*_: activity of both LTZ and interferent.

For further confirmation, interferent solutions covering the working ranges of each electrode were prepared for the three sensors, potentials were recorded, and a calibration graph was plotted for each sensor. However, the primary ion may leach from the membrane and hence exaggerates the response to an ion that interferes weakly. To eliminate the bias caused by leaching, potentials of interferent solutions were recorded using membranes that had never been in contact with the primary ion. Resulting calibration plots represent the unbiased selectivity^48,49^.

#### Determination of LTZ in Femara® tablets

One tablet (2.5 mg/tab) was ground and transported to a volumetric flask (50 mL) where it was mixed with 1:4 HCl solution till dissolved; producing a final concentration of 1.75 × 10^–4^ M, then potential was measured. The procedure was repeated for 3 tablets and concentrations were calculated from the corresponding regression equation of each electrode. The standard addition technique was also carried out for validity testing of the suggested sensors.

#### Determination of LTZ in human plasma

Proper dilution of working solutions using plasma was performed to prepare plasma samples (three concentrations were prepared, including C_max_). For protein precipitation, 2.00 mL of acetonitrile was added then solution was mixed and centrifuged for 15 min at 6000 rpm. Supernatant was collected, evaporated, and then reconstituted with 1:4 HCl solution. EMF was recorded after immersion of the sensor in the prepared samples and the corresponding concentrations were calculated.

Same procedure was carried out without prior protein precipitation and results were compared.

## Results and discussion

The major objective of the present work is to create potentiometric electrodes for on-site LTZ measurement and to simplify their use for clinical monitoring. Three solid contact electrodes were created and compared. The use of conducting polymers as internal contact in solid contact electrodes typically shows enhanced sensitivity. The study emphasized the positive impacts of utilizing solid contact ion-selective electrodes for assessing LTZ in plasma rather than time-consuming, expensive conventional methods. The proposed modified sensors outperformed the previously reported PVC and carbon paste electrodes in terms of lower detection limits and better sensitivity. The suggested PANI sensor shows a lower detection limit than the previously prepared LTZ-PM-MgONPs sensor, and a better response time compared to LTZ-PM-MgONPs and LTZ-PM-CuONPs sensors^23^.

### Characterization of polyaniline nanoparticles

#### Characterization of polyaniline nanoparticles using UV–Visible spectroscopy

The synthesized nanoparticles were investigated regarding their electronic states in two forms: The conducting emeraldine salt (ES), and the insulating emeraldine base (EB). Typical spectra of both forms were observed as shown in supplementary Fig. S1 online. There are two distinct absorption bands for the conducting ES, the first appears at 420 nm which is due to the π–π* transition of the benzenoid rings. A second band appears around 850 nm which is due to the π-Polaron transition. Upon being treated with 0.5 M NaOH, PANI nanoparticles transform into the insulating EB where a shift of the absorption peaks to 330 and 615 nm was observed due to the π–π* transition of the benzenoid ring and the exciton band of the EB form, respectively^50^.

#### Characterization of polyaniline nanoparticles using dynamic light scattering (DLS)

The prepared particles were found to be in the nano range with a mean hydrodynamic size of 102.10 nm as illustrated in supplementary Fig. S2 online.

### Performance elements of the fabricated sensors

As widely known, the main components of an electroactive membrane are PVC matrix, plasticizer, and the sensing material. The fabricated sensors were based on the stable host–guest complex formed between the drug cation and the selected ionophore (TBCAX-8). LTZ has two benzonitrile groups and a tertiary amine group that are converted to cations in acidic media. Upon the addition of tetraphenyl borate to the LTZ-ISM as a cation exchanger, a 1:3 association complex resulting from the interaction of LTZ and tetraphenylborate inside the ionophore is formed. This complex was utilized as the sensing element in the three developed electrodes. The incorporation of an extra ion-to-electron transducer layer between the solid contact and the ion-selective membrane was attempted using graphene nanocomposite and polyaniline nanoparticles in the second and third electrodes; respectively to enhance the reproducibility and sensitivity of the electrode and limit the uptake of water.

In order to assess the influence of membrane composition on the electrochemical response of electrodes, the impact of the used modifiers; GNC and PANI NPs was investigated and compared to the plain sensor.

The electrochemical performance of TBCAX-8, GNC and PANI NPs based sensors (1, 2 and 3; respectively) was studied following the IUPAC recommendations and data is summarized in Table 1.

**Table 1:** Performance characteristics of the fabricated sensors.

Parameter	Sensor 1	Sensor 2	Sensor 3
Slope* (mV.decade^-1^) ± SD	19.90 ± 0.40	20.10 ± 0.30	20.30 ± 0.40
Intercept	362.20	187.00	482.60
Correlation coefficient (r)	0.9997	0.9998	0.9999
Linearity range (M)	1.00 × 10^–5^–1.00 × 10^–2^	1.00 × 10^–6^–1.00 × 10^–2^	1.00 × 10^–8^–1.00 × 10^–3^
LOD (M)	9.23 × 10^–6^	9.45 × 10^–7^	4.04 × 10^–9^
Response time (sec.)	35–45	20–30	10–12
Lifetime (days)	45	60	15
Working pH range	2–5	2–5	2–5
Temperature (°C)	25	25	25
Accuracy (mean ± SD)	100.00 ± 0.80	99.70 ± 0.70	100.00 ± 0.60
Repeatability (%RSD)	0.68	0.81	0.26
Intermediate precision** (%RSD)	0.85	1.01	0.38

* Average of 3 determinations.

**Intermediate precision studied by analysis of different concentration levels of LTZ on three different days.

**Figure 2:**
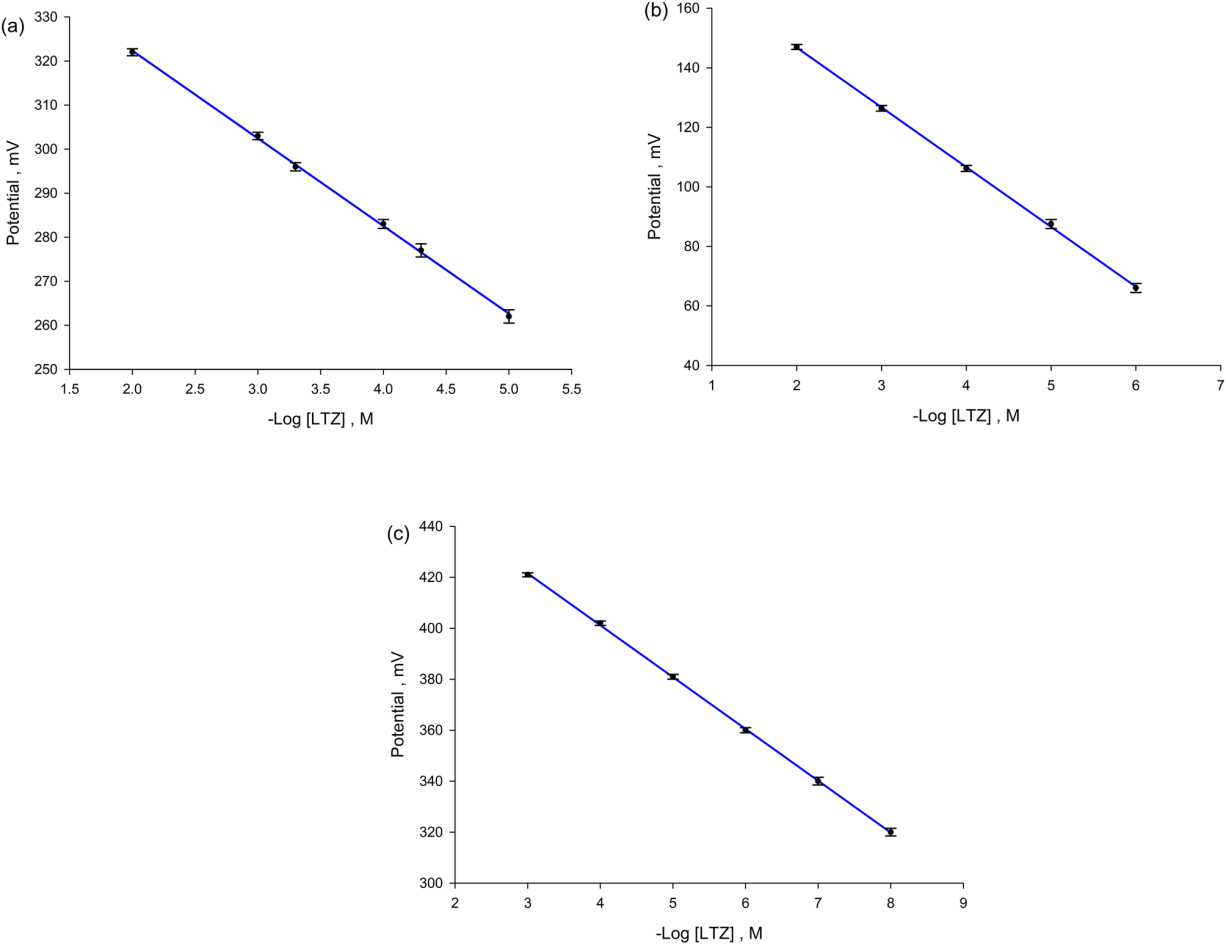
Profile of the potential in mV versus − Log [LTZ] for (**a**) sensor 1, (**b**) sensor 2 and (**c**) sensor 3.

Calibration curves (Fig. 2) were constructed for the proposed sensors where all sensors showed linear responses. Detection limits shown in Table 1 were calculated from the intersection of the two extrapolated segments of the calibration plot.

Slopes of calibration plots were 19.90, 20.10, 20.30 for sensors 1, 2 and 3; respectively. These values mimic a nearly sub-Nernstian slope of a trivalent cation and strongly support the deducted ion pair ratio. This agrees with the reported letrozole sensor^22^, where the best sensor tested was a nanocomposite carbon paste which showed a slope of 19.7 mV/decade.

**Figure 3:**
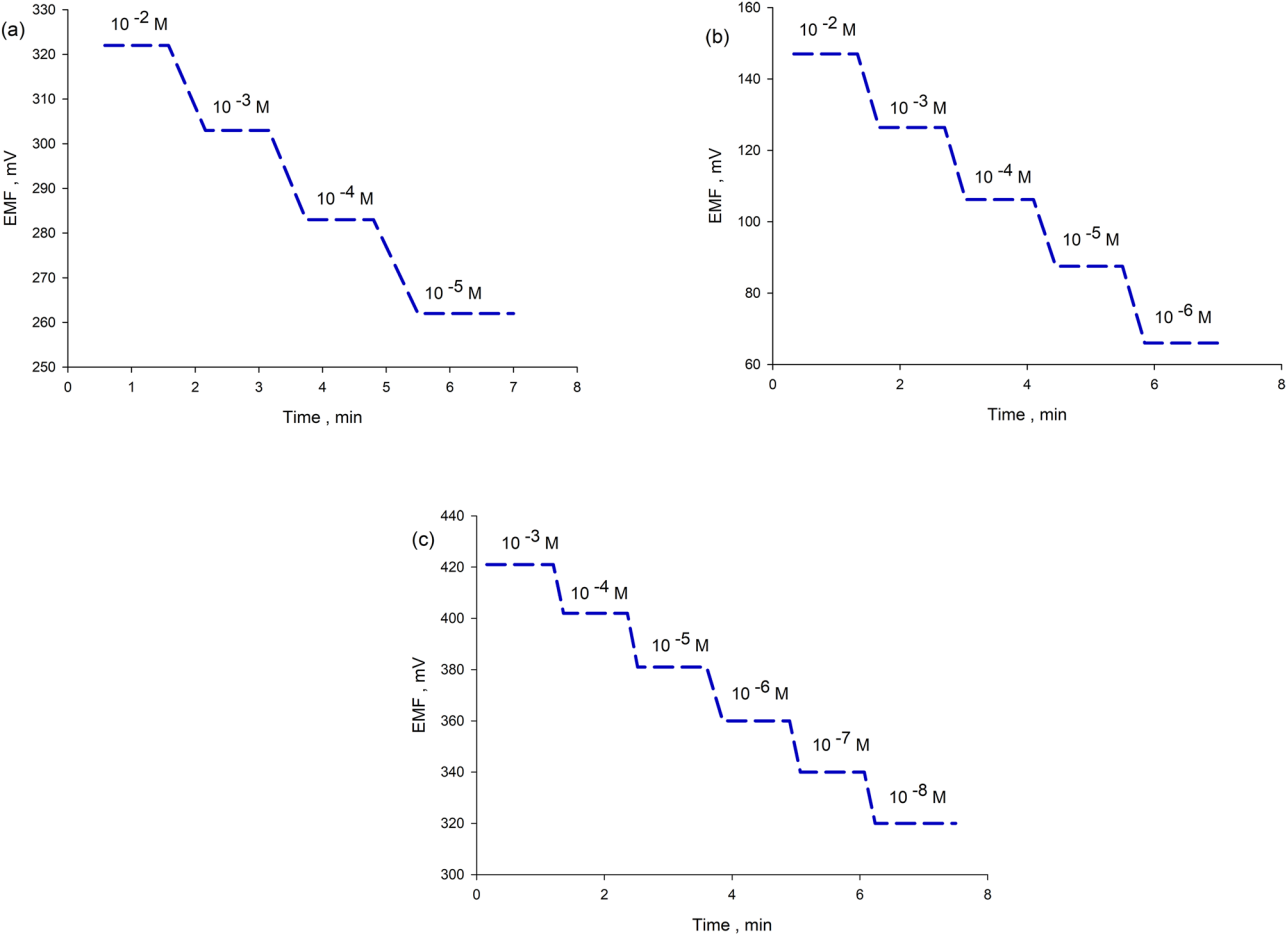
Dynamic response time of the proposed sensors towards step changes in LTZ concentration (**a**) sensor 1 (**b**) sensor 2 (**c**) sensor 3.

Modified electrodes showed relatively faster response than the conventional one, the time needed for the electrode to reach a potential value within ± 1 mV of the final equilibrium value was 25, 11 s for sensors 2 and 3 versus 40 s for sensor 1 as shown in Fig. 3. We suggest that this is due to larger contact area between the solution and the nano-sized membrane resulting in faster ion to electron transition.

The potential readings were found to be stable within 3 mV from one day to another and the slopes were almost the same along the lifetime period of each electrode.

Regarding the electrode’s stability, sensor 2 was the most stable, reaching a life span of 60 days. The introduction of graphene nanoplatelets into the sensing membrane caused an increase in the specific surface area, which in turns enhanced the stability of the electrical signal and lessened the likelihood of potential drift due to raised double layer capacitance of the modified electrode.

Generally, sensor 3 (PANI electrode) showed superior results in terms of linearity, response time and LOD. This might be due to enhanced electrical conductivity, but it has the drawback of a short lifetime (15 days) compared to other fabricated sensors. Upon comparing our modified sensors to previously fabricated LTZ sensors, we observed enhanced sensitivity and response times. LOD values of GNC and PANI sensors reached 9.45 × 10^–7^, 4.04 × 10^–9^ M versus 3.00 × 10^–6^, 1.00 × 10^–6^, 5.90 × 10^–9^ M for PVC, CPE^22^, LTZ-PM-MgONPs sensors^23^; respectively. Response times of 20, 15, 45, and 30 s were recorded for PVC, CPE, LTZ-PM-MgONPs, and LTZ-PM-CuONPs; respectively. Thus, the developed PANI sensor comes across as the fastest with a response time of 11 s, which maximizes its throughput where a higher number of samples can be processed over short time periods.

### Effect of pH

**Figure 4:**
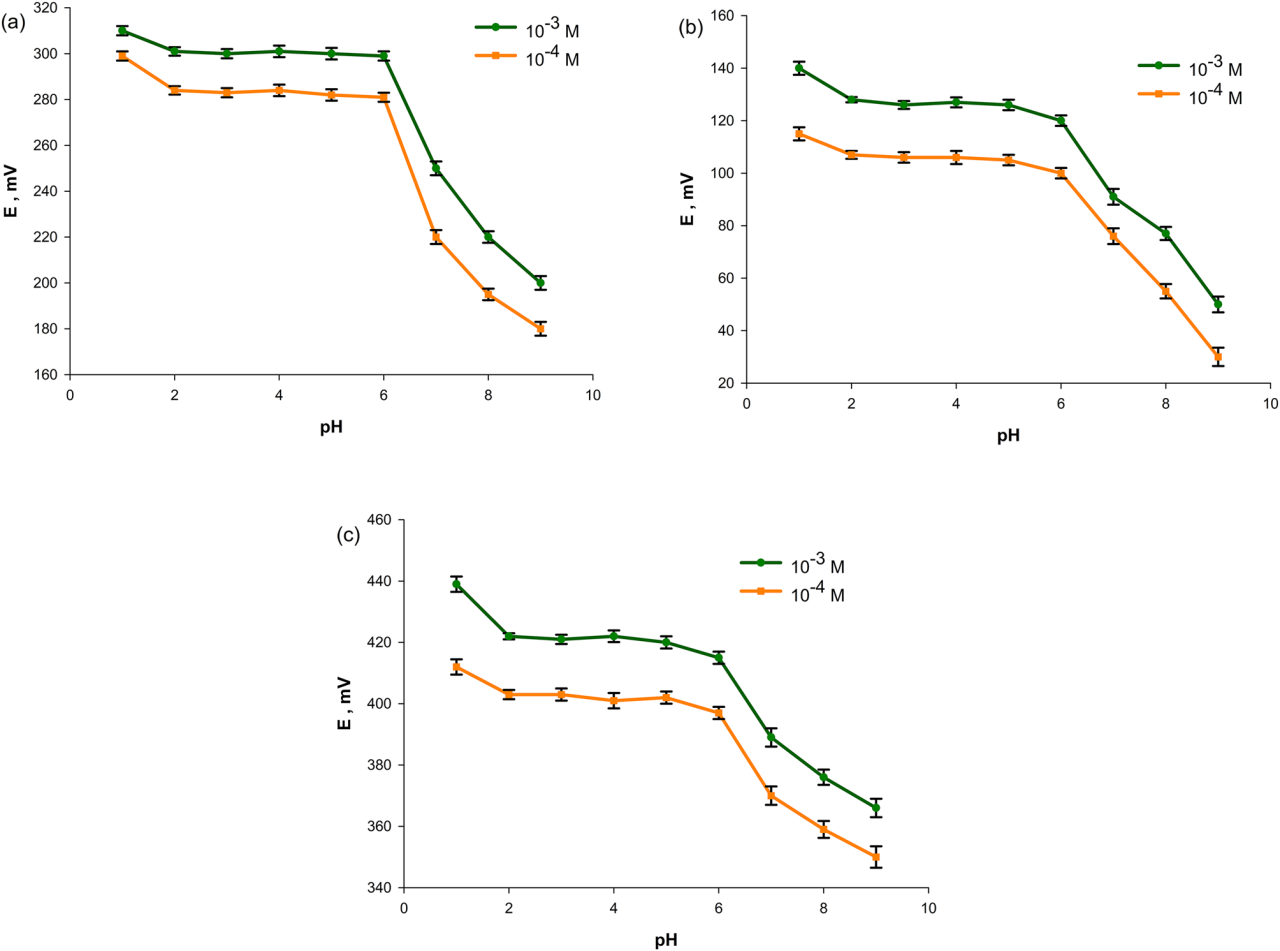
Effect of pH on the response of the proposed sensors (**a**) sensor 1 (**b**) sensor 2 (**c**) sensor 3.

Upon exploring how the response of the fabricated electrodes is affected by changing pH values, it was found that the best pH value was within 2–5 for sensors 1, 2 and 3 where the obtained potentials were almost constant. LTZ has pKa values of 4.4, 5.4 corresponding to the triazole ring protons and the dibenzonitrile group; respectively^51^. In the working pH 2, the ionized form of LTZ predominates. This explains the above behavior and coincides with the previously obtained effective pH ranges for different LTZ sensors^22,23^. Noisy potentials were noticed on measuring above and below this range as (Fig. 4). This may be due to interference from hydroxyl and hydronium ions, where their concentrations become relatively higher than the primary ion. Being basic, precipitation of the drug base may occur at higher pH values. Also, possible degradation of the drug at these high pH values may account for the resulting potential changes^52^.

### Effect of temperature

No significant change in the sensors’ potential values was observed during measurements at elevating temperature. Parallel calibration plots were obtained at all investigated temperatures.

### Selectivity

In the presence of possible interferents, selectivity of the three suggested electrodes was assessed using separate solution method, where a fixed concentration of the principal ion and interferents (10^–3^ M) was measured and selectivity coefficient values (K_pot LTZ, I_) were calculated. For each interferent, calibration plots of each sensor were constructed, and a non-Nernstian slope was obtained for all of them. To confirm the selectivity of the proposed sensors, membranes not in previous contact with LTZ solutions were implemented as recommended by the IUPAC^53^. Since it is quite challenging to interact with the membrane when the principal ion is present, this was done to enable any interferent ion that might show a Nernstian response to do so. The obtained results displayed in supplementary Fig. S3 online confirmed the very good selectivity of the proposed sensors towards LTZ while many interfering ions and co-administered drugs are present.

Sensor 3 showed the highest selectivity with significantly lower values of selectivity coefficients compared to the other sensors as shown in Table 2. However, the proposed sensors turned out to be the most selective amongst all previously prepared sensors for LTZ assay with selectivity coefficient values of 3.98 × 10^–6^, 1.86 × 10^–13^ for sodium ion as an example versus 3.16 × 10^–4^, 2.50 × 10^–4^, 4.80 × 10^–4^, 9.20 × 10^–5^ for PVC, CPE, LTZ-PM-MgONPs and LTZ-PM-CuONPs sensors; respectively. Same applies for other interferents mentioned below.

**Table 2:** Potentiometric selectivity coefficients of the proposed sensors.

Interferent	Selectivity coefficient		
	Sensor 1	Sensor 2	Sensor 3
Na^+^	3.24 × 10^–4^	3.98 × 10^–6^	1.86 × 10^–13^
K^+^	2.88 × 10^–5^	1.94 × 10^–6^	5.01 × 10^–13^
NH_4_^+^	1.82 × 10^–3^	7.07 × 10^–7^	1.00 × 10^–14^
Ca^++^	3.31 × 10^–7^	6.17 × 10^–11^	1.40 × 10^–15^
Mg^++^	1.86 × 10^–6^	1.23 × 10^–10^	1.09 × 10^–18^
Starch	1.26 × 10^–6^	2.50 × 10^–7^	1.34 × 10^–13^
Lactose	5.86 × 10^–11^	3.98 × 10^–15^	3.96 × 10^–21^
Clomiphene citrate	7.31 × 10^–3^	8.62 × 10^–5^	2.07 × 10^–7^
Metformin HCl	4.55 × 10^–4^	2.76 × 10^–6^	1.28 × 10^–9^
Tamoxifen	2.07 × 10^–2^	4.33 × 10^–5^	1.35 × 10^–8^

### Water layer test

Due to the multi-layer consistency of SC-ISEs, a common issue needs to be investigated to ensure the integrity of these layers and the absence of any “aqueous layer” between them. Whenever a water layer is formed between the solid contact electrode and the ion-selective membrane, a “potential drift” results causing unsatisfying electrode reproducibility. Water layer test has become an essential step in evaluating the performance of SC-ISEs^54^. Water layer testing starts with recording the electrode potential in a solution of 1.00 × 10^–4^ M of LTZ after full equilibration for 1 h. Then the electrode is inserted in a solution of a higher concentration of the interfering ion and potential is recorded for 2 h, and eventually transferred back to the drug solution where potential is recorded again for 5 h.

Clomiphene citrate is commonly administered along with letrozole for ovulation induction in patients with PCOS. Thus, it was selected for this test.

**Figure 5:**
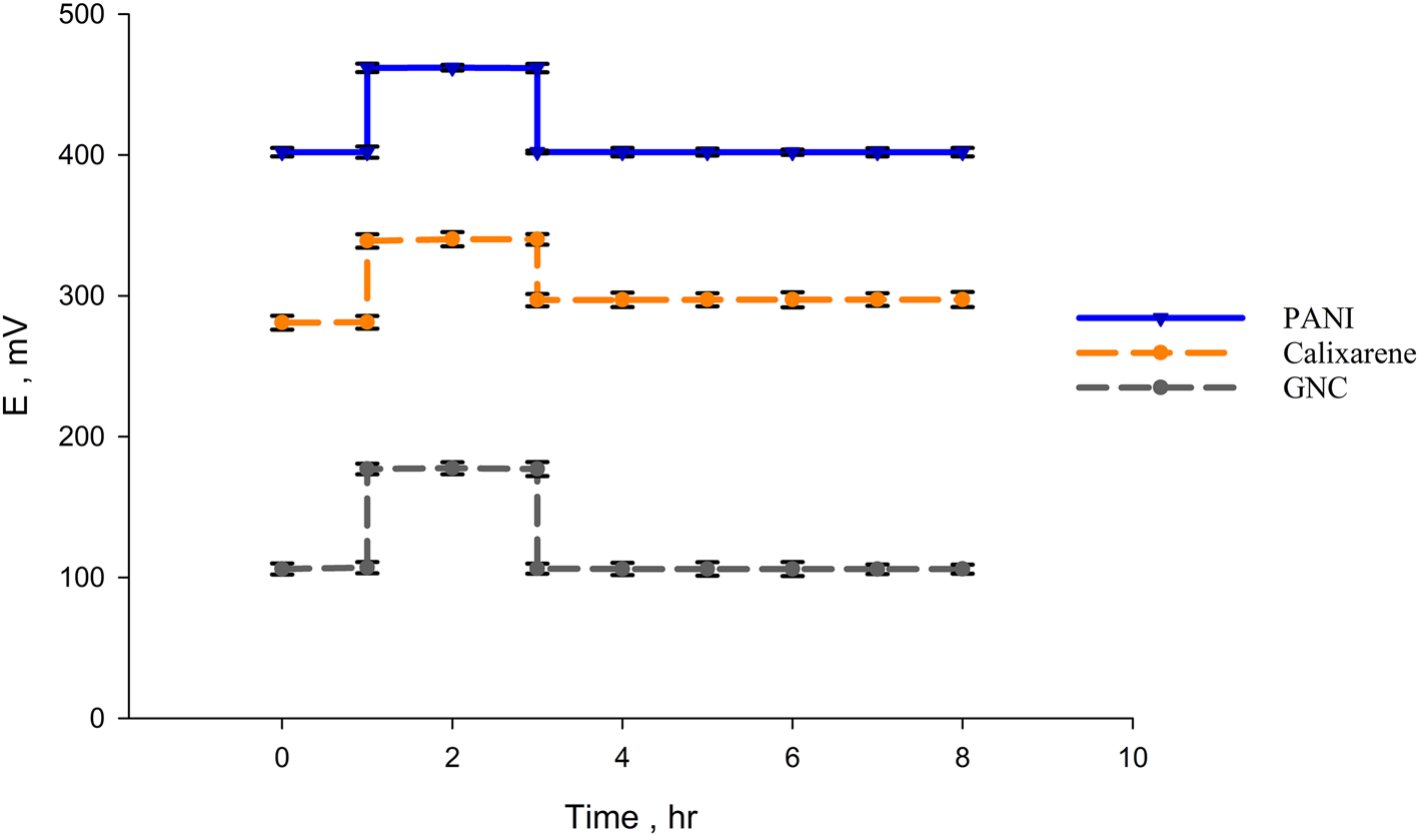
Potentiometric water layer test.

After applying these steps on our proposed electrodes using clomiphene citrate as the interferent^55^, a slight drift was noticed in sensor 1, indicating that a water layer was formed between graphite and TBCAX-8 based membrane. No signs of potential drift were noticed in sensors 2 & 3. Thus, modifications made using GNC and PANI helped in preventing the water layer formation as illustrated in Fig. 5.

### Determination of LTZ in Femara® tablets

Since most excipients used in formulating drugs exhibited no interference, no prior treatment or extraction was made before carrying out the analysis. All developed sensors were successfully employed in LTZ direct determination in Femara tablets (Table 3) and after applying standard addition (Table 4).

**Table 3:** Direct determination of LTZ in its dosage form using the proposed sensors.

Dosage form Femara tablets claimed conc. (M)	Sensor 1	Sensor 2	Sensor 3
	Recovery% ± S.D*	Recovery% ± S.D*	Recovery% ± S.D*
1.75 × 10^–4^	96.50 ± 1.39	98.69 ± 0.69	99.64 ± 0.40

*Average of 3 determinations.

**Table 4:** Standard addition technique for the determination of LTZ in Femara® tablets by the proposed sensors.

Sensor 1			Sensor 2			Sensor 3		
Added (M)	Found	Recovery%	Added (M)	Found	Recovery%	Added (M)	Found	Recovery%
1.00 × 10^–4^	9.64 × 10^–5^	96.40	1.00 × 10^–4^	9.85 × 10^–5^	98.50	1.00 × 10^–4^	1.00 × 10^–4^	100.00
2.00 × 10^–4^	1.94 × 10^–4^	97.00	2.00 × 10^–4^	1.97 × 10^–4^	98.50	2.00 × 10^–4^	1.99 × 10^–4^	99.50
3.00 × 10^–4^	2.89 × 10^–4^	96.30	3.00 × 10^–4^	2.95 × 10^–4^	98.30	3.00 × 10^–4^	2.99 × 10^–4^	99.67
Mean ± S.D		96.57 ± 0.38	Mean ± S.D		98.43 ± 0.12	Mean ± S.D		99.72 ± 0.25

### Determination of LTZ in human plasma samples

In this study, only sensor 3 (PANI) was implemented, as sensors 1, 2 linear ranges didn’t cover maximum plasma LTZ concentration^7,56^. Evaluation of the effect of plasma protein precipitation was carried out and better results were obtained in case of plasma protein precipitation prior to analysis although acceptable results were obtained when no plasma protein precipitation was carried out as shown in Table 5.

**Table 5:** Determination of LTZ spiked human plasma samples by sensor 3.

Added (M)	No Protein precipitation		Protein precipitation with ACN	
	Found (M)	Recovery%	Found (M)	Recovery%
6.00 × 10^–7^	5.37 × 10^–7^	89.50	5.76 × 10^–7^	96.00
1.75 × 10^–7^	1.54 × 10^–7^	88.00	1.67 × 10^–7^	95.43
1.00 × 10^–7^	8.84 × 10^–8^	88.40	9.63 × 10^–8^	96.30
	Mean ± S.D	88.63 ± 0.78	Mean ± S.D	95.91 ± 0.44

### Greenness assessment

Owing to the current conditions of climate change and its accompanied threats, there has been a growing global trend to develop green analytical methods to help reduce and/or eliminate the implementation and production of hazardous substances^57,58^. Green analytical chemistry (GAC) is a green chemistry branch where developing analytical procedures that are safer to humans and environment with no impact on the analytical figures of merit is the main focus^59^. Principles of GAC are applicable to different analytical techniques and most recent research show huge efforts in this field ^60–62^. Electrochemistry is considered a relatively simple technique where direct sample measurement is possible without prior treatment or collection steps. Several tools were developed to be used in assessing the greenness of analytical methodologies, as Analytical Eco-scale, The Green Analytical Procedure Index (GAPI), National Environmental Methods Index (NEMI), and Analytical GREEnness metric (AGREE)^59^. Analytical Eco-scale and Analytical GREEnness metric were adopted in this study.

#### Analytical eco-scale

On the Eco-scale, it is presumed that the optimal method of analysis would score 100 and each deviation from this ideality in the proposed method parameters is assigned some penalty points that lower the total score. Results are explained as follows; a score exceeding 75: excellent green analysis, above 50: acceptable green analysis, and below 50: inadequate green analysis. Parameters assessed include the type and amount of used reagents, energy consumption of different electric equipment, amount of waste and its treatment, and finally occupational hazard^63–66^.

Upon evaluation of our proposed method, it reveals to be an excellent green analytical method as depicted in supplementary Table S1 online.

#### Analytical GREEnness metric (AGREE)

A comprehensive metric system where the 12 principles of green analytical chemistry are considered while assessing the greenness of the method. The final score is presented in the middle of a segmented pictogram where each criterion is assigned a color and a weight corresponding to the performance of the analytical procedure in it. A final score closer to 1 with a darker green color in the middle of the pictogram and in each segment signifies a greener procedure^66^. The proposed method achieved a score of 0.81 with a green color in the center (see supplementary Fig. S4 online), so it’s considered to be a green method of analysis.

#### Statistical analysis

To examine the validity of the proposed method, a statistical comparison was performed between the proposed method and a reported one. Student^'^s t test and variance ratio F test were conducted and summarized in Table 6. The calculated t and F values were less than theoretical ones at *p* = 0.05, indicating there is no statistically significant difference between the reported and the proposed method.

**Table 6:** Statistical comparison between the results of analysis of LTZ by the proposed method and a reported RP-HPLC method.

Parameter	Proposed method			Reported method**
	Sensor 1	Sensor 2	Sensor 3	
Mean	100.00	99.70	100.00	100.16
SD	0.80	0.70	0.60	0.90
Variance	0.64	0.49	0.36	0.80
n	5	5	5	5
Student t-test *(2.306)	0.12	0.84	0.54	–
F *(6.388)	1.25	1.63	2.22	–

*The theoretical values of t and F at *p* = 0.05.

**The reported method^66^: RP-HPLC method using a C_18_ column, (50 mm × 2.1 mm, 1.7 µm particle size). The mobile phase was composed of acetonitrile, water, and methanol eluted isocratically at a ratio of (50:35:15, v/v/v) at a flow rate of 1.5 mL/min.

## Conclusions

In the current work, we propose three novel simple, green sensors and their application in the potentiometric determination of letrozole in pharmaceutical dosage form and human plasma. TBCAX-8 was utilized as an ionophore in constructing the potentiometric sensors due to its extraordinary ability to complex organic ammonium ions. Further modifications with graphene nanocomposite and polyaniline nanoparticles were made, which were extremely beneficial in terms of decreasing response time and boosting the sensors' sensitivity and selectivity towards LTZ.

### Supplementary Information

Supplementary Information.

## Data Availability

The datasets generated or analysed during the current study are included in the manuscript. Further details and related data are available from the corresponding author upon reasonable request.
