# Supplementary material for: A comparative study of green solid contact ion selective electrodes for the potentiometric determination of Letrozole in dosage form and human plasma
Source: Sci Rep. 2023 Nov 18;13:20187. doi: 10.1038/s41598-023-47240-3 (PMC10657372; doi:10.1038/s41598-023-47240-3)
Supplement: Supplementary file 1 — Supplementary Information. [file 41598_2023_47240_MOESM1_ESM.docx]

**Supplementary file for: A comparative study of green solid contact ion selective electrodes for the potentiometric determination of Letrozole in dosage form and human plasma**

Sherin M. Alqirsh^a^, Nancy Magdy^a^, Maha F. Abdel-Ghany^a^, Noha F. El Azab^a^

^a^Pharmaceutical Analytical Chemistry Department, Faculty of pharmacy, Ain Shams University, Organization of African Unity Street, Abasia, 11566, Cairo, Egypt.

***Correspondence:** [**sherinmohamed.mohamed@pharma.asu.edu.eg**](mailto:sherinmohamed.mohamed@pharma.asu.edu.eg)


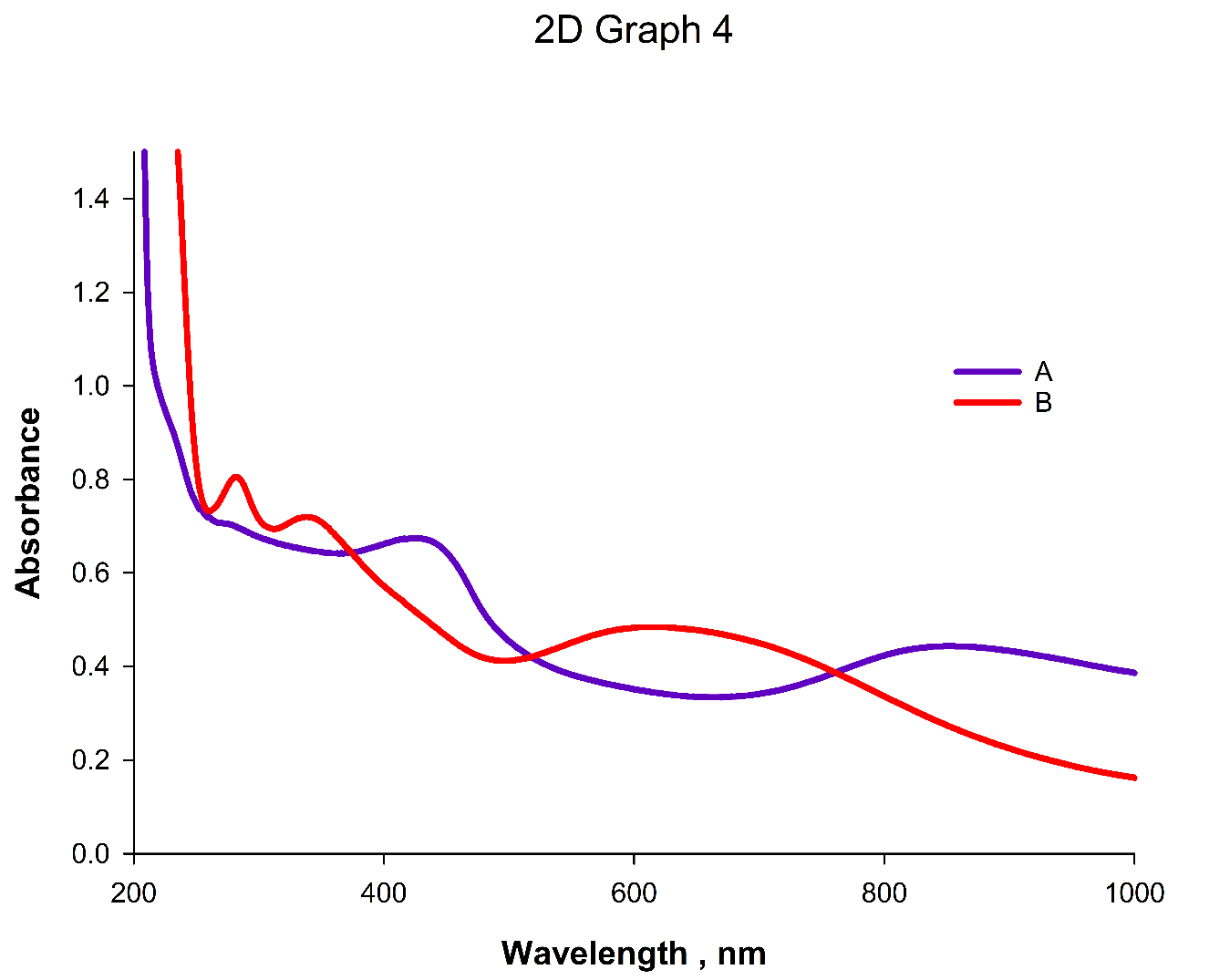


**Fig.S1.** UV-VIS characterization of PANI nanoparticles in (A) ES form and (B) EB form.


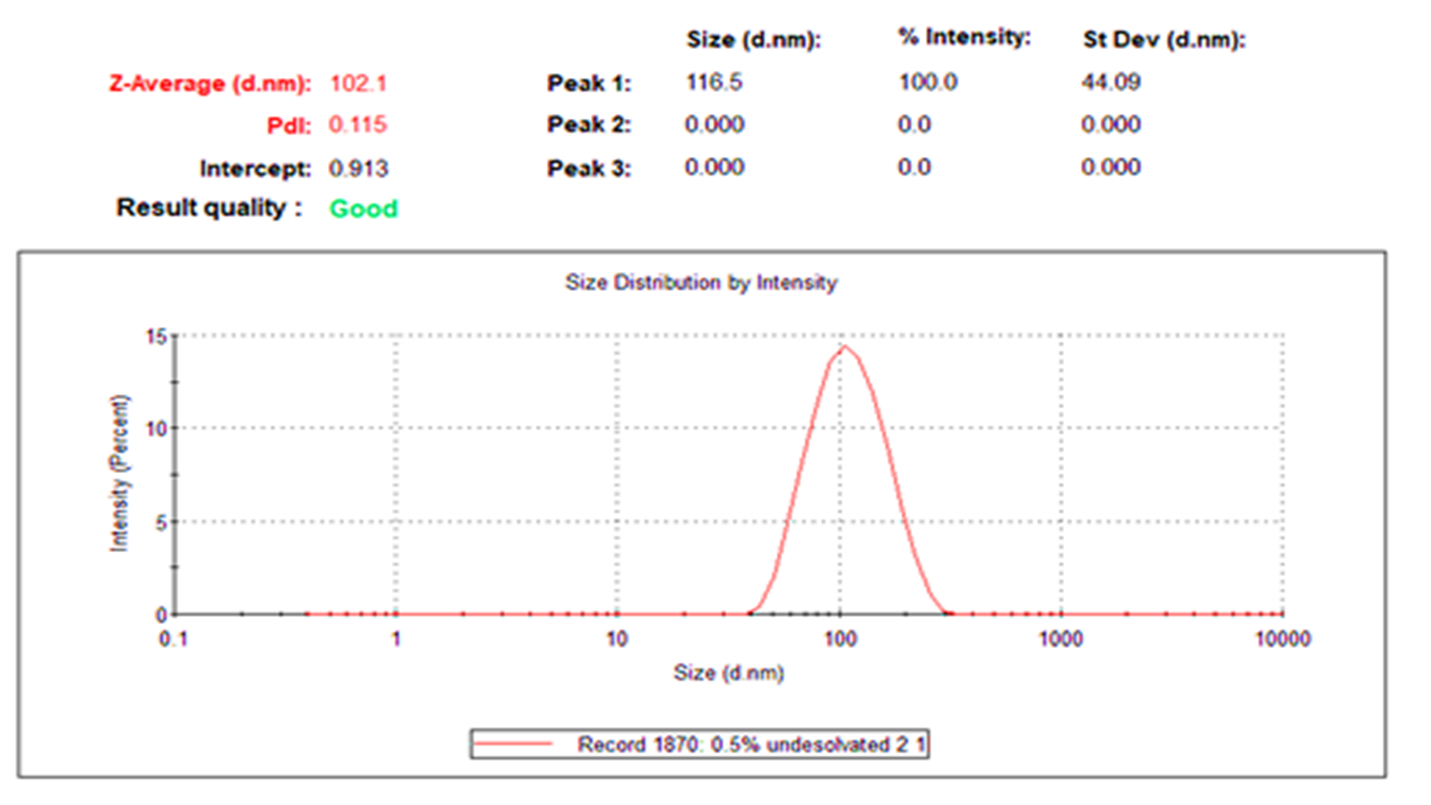


**Fig.S2.** Particle size of PANI NPs using diffraction light scattering (DLS).


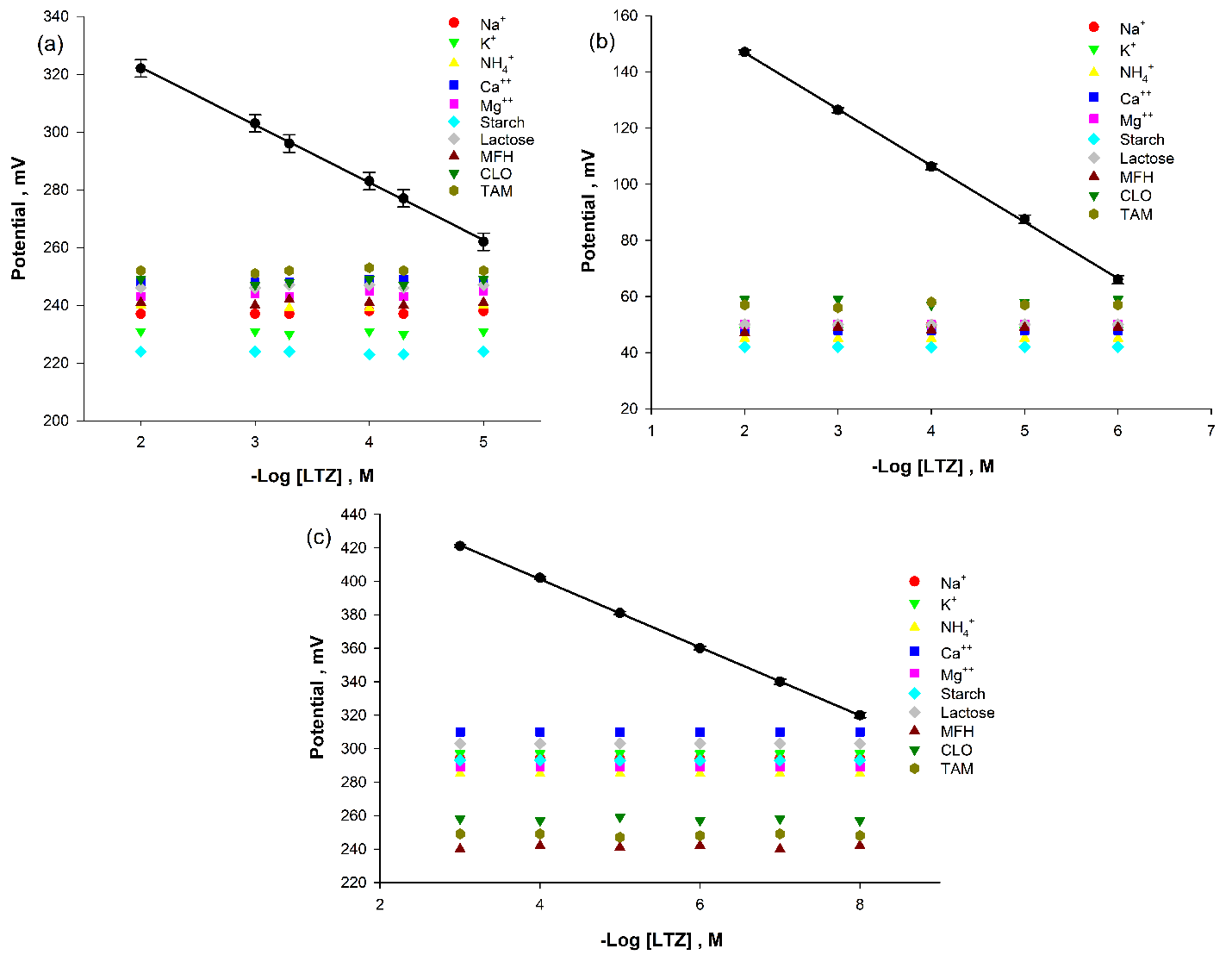


**Fig.S3.** Determination of selectivity coefficients for the fabricated sensors (a) sensor 1 (b) sensor 2 (c) sensor 3, towards possible interfering ions and co-administered drugs.


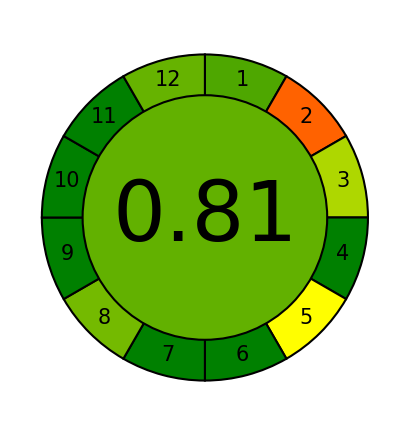


**Fig.S4.** Greenness assessment of the proposed method using AGREE software.

**Table S1. The penalty points for the proposed potentiometric method according to Analytical Eco-scale.**

| **Parameters** | **Penalty points for the proposed sensors (PPs)** |
| --- | --- |
| - **Reagents:**   Diluted HCl solution  Water  Polyvinyl chloride  Di-octyl phthalate  Sodium tetraphenylborate  Tetrahydrofuran  Calix-8-arene  Graphene  Xylene  Aniline  Sodium dodecyl sulfate  Ammonium persulfate  Acetonitrile   - **Instruments:**   Energy  Occupational hazard  Waste  **Total PPs**  **Analytical Eco-scale score** | 3  0  0  0  1  2  0  0  2  2  2  2  1  0  0  0  **∑ 15**  **85** |
